# Superannuation and the Young Nurse

**Published:** 1921-04-23

**Authors:** 


*To the Editor of* The Hospital.

### Superannuation and the Young Nurse.

*To the Editor of* The Hospital.

Sir,—I was interested to notice that in article six, "The Pay of the Nurse," in your last issue you deal with the question of superannuation. On completing training in a provincial hospital I took a post as ward sister in a Poor-law hospital; I was young, and the world before me. One morning the matron wished to see me in her office, and I was asked by her whether or not I wished to contract out of the Poor-law Officers' Superannuation scheme. I had never heard of it before, and the question put was, "Do you intend to remain Poor-law nursing all your life?" At the age of twenty-six I had no such intention. Neither did I desire my salary of £2 10s. per month to be depleted in any way, so I signed the paper, and that was the end of that. This was a "pre-war" incident, and I am still Poor-law nursing. Many of my colleagues, who like myself are beginning to think seriously about the future, remember a similar incident, and, like myself, regret that step. Yet who is to blame? It has always struck me as being singular that matrons have never taken the trouble to explain to young nurses the real benefits of the superannuation scheme, but rather to sneer at it, and to deprecate that they themselves have compulsorily to contribute.—Yours, etc.,

Celtic.

